# The Use of Stents in Children with Nasolacrimal Duct Obstruction Requiring Surgical Intervention: A Systematic Review

**DOI:** 10.3390/ijerph17031067

**Published:** 2020-02-07

**Authors:** Evelyn Li Min Tai, Yee Cheng Kueh, Baharudin Abdullah

**Affiliations:** 1 Department of Ophthalmology, School of Medical Sciences, Health Campus, Universiti Sains Malaysia, Kubang Kerian 16150, Malaysia; daileid@yahoo.com; 2 Hospital Universiti Sains Malaysia, Universiti Sains Malaysia, Kubang Kerian 16150, Malaysia; baharudin@usm.my; 3 Unit of Biostatistics & Research Methodology, School of Medical Sciences, Health Campus, Universiti Sains Malaysia, Kubang Kerian 16150, Malaysia; 4 Department of Otorhinolaryngology, School of Medical Sciences, Health Campus, Universiti Sains Malaysia, Kubang Kerian 16150, Malaysia

**Keywords:** lacrimal intubation, endoscopic dacryocystorhinostomy, external dacryocystorhinostomy, surgical intervention, balloon dacryoplasty, dye disappearance test, children

## Abstract

Nasolacrimal duct obstruction (NLDO) is the most common cause of childhood epiphora. It is managed conservatively in the first year of life, after which surgical treatment is classically based on a stepwise paradigm of probing, intubation, and dacryocystorhinostomy. This systematic review aims to present the current role of intubation in the management of children with NLDO requiring surgical intervention. A search for English-language articles from the electronic databases PubMed, SCOPUS, and the COCHRANE library was conducted over a period of five months in accordance with the Preferred Reporting Items for Systematic Reviews and Meta-Analyses guidelines and the Cochrane Handbook. The following keywords were used to aid retrieval: stents, children, lacrimal intubation, endoscopic dacryocystorhinostomy, external dacryocystorhinostomy, NLDO, dacryocystitis, congenital, acquired. The primary outcome was defined as the success of the intervention, determined by resolution of symptoms and patency of the lacrimal anatomy confirmed by the fluorescein dye disappearance test or syringing. Secondary outcomes included the presence of complications. A total of 144 articles were identified; of these, 35 fulfilled the study criteria. The majority of the included studies involved lacrimal intubation alone, followed by intubation as an adjunctive procedure to balloon dacryoplasty and dacryocystorhinostomy. The overall success rate of these procedures ranged from 41.1% to 100%. Post-operative complications were reported in 65.7% of the included studies. Lacrimal intubation was most commonly performed as a primary procedure in children with NLDO, with high success rates. The main complication was stent dislodgement. There is lack of evidence regarding the benefit of intubation over probing as primary treatment of congenital NLDO. In the absence of high-quality evidence, the decision of whether to perform lacrimal intubation in children with NLDO requiring surgical intervention depends on clinical judgement and other low-level evidence, such as observational non-randomised trials.

## 1. Introduction

Nasolacrimal duct obstruction (NLDO) is the most common cause of childhood epiphora [1]. Failed canalisation of the distal nasolacrimal duct, associated with a membranous obstruction at the level of Hasner’s valve, is the main cause of congenital NLDO; acquired causes of NLDO include infections and traumatic obstructions [2]. Congenital NLDO is managed conservatively in the first year of life, usually resolving spontaneously, coincident with elongation and volume expansion of the nasolacrimal duct [3,4,5]. Epiphora which persists after the age of one year old may require surgical intervention, as the success rates of conservative treatment decline with increasing age [6]. 

The surgical treatment of NLDO in children is classically based on a stepwise paradigm, with probing as the primary procedure, followed by balloon catheter dilation [7]. Intubation has traditionally been reserved for congenital NLDO refractory to other measures [7,8,9]. Intubation involves the placement of a stent within the nasolacrimal duct to prevent re-closure of the membranous obstruction. With the advent of technologically superior instrumentation and surgical skills, lacrimal intubation is not only an increasingly popular alternative to dacryocystorhinostomy (DCR) for cases which fail conservative management and probing, but also serves as an adjunct during balloon dacryoplasty [9] and DCR [10,11]. This systematic review aims to present the current role of intubation in the management of children with NLDO requiring surgical intervention.

## 2. Materials and Methods

### 2.1. Search Strategies

The search was conducted over a period of 5 months (November 2018 to March 2019) in accordance with the Preferred Reporting Items for Systematic Reviews and Meta-Analyses (PRISMA) guidelines [12] and the Cochrane Handbook [13] when appropriate. The literature search was conducted by searching for English-language articles from the electronic databases PubMed, SCOPUS, and the COCHRANE library. The following keywords were used either individually or in combination to aid in retrieving the articles: stents, children, lacrimal intubation, endoscopic DCR (EDCR), external DCR (extDCR), NLDO, dacryocystitis, congenital, acquired. Literature for inclusion in the review was restricted to the period of 1997 to 2019 in order to keep the information as relevant and up to date as possible.

### 2.2. Eligibility Criteria

Articles were included in the systematic review if they fulfilled the following eligibility criteria: (1) prospective comparative design (e.g., randomised and non-randomised controlled trials (RCT), cohort study), retrospective with comparative group design (e.g., case-control, cross-sectional), retrospective or prospective non-comparative design (e.g., case series, before and after study); (2) included participants were less than 18 years of age; (3) intubation was part of the management of NLDO. Articles were excluded if they were (1) case reports; (2) abstract only studies; (3) published in a language other than English.

### 2.3. Study Outcomes

The primary outcome was defined as the success of the intervention, determined by the resolution of symptoms and the patency of the lacrimal anatomy confirmed by the fluorescein dye disappearance test, syringing or irrigation of the nasolacrimal duct. The secondary outcome was the presence of complications.

### 2.4. Screening and Data Extraction

The authors selected the studies according to the predetermined inclusion and exclusion criteria for this systematic review. This was done by reading the abstracts and/or the full articles. A standardised data extraction form was developed by the authors and used in the present study. The variables extracted from the studies included study location (country), number of patients, age, gender, intervention procedure, duration of tube removal, duration of follow up, overall successful outcome, and post-operative complications. Data extraction from the included studies was done by two authors independently. Any discrepancy between the two authors with respect to the data extracted were discussed. When disagreements remained, a third author was consulted for his/her opinion and decision.

### 2.5. Quality Assessment

The quality assessment of the included studies was conducted by using the Effective Public Health Practice Project (EPHPP) checklist [14]. The EPHPP has been widely used in assessing the quality of public health intervention studies of varying study designs [15,16,17]. The EPHPP checklist has six components of assessment of study methodology; selection bias, study design, confounders, blinding, data collection methods, withdrawal and dropouts. These components were scored as either weak, moderate, or strong. The overall quality rating for each included study was also scored as either weak, moderate, or strong. An overall quality rating of ‘strong’ was assigned when there were no weak ratings, ‘moderate’ was assigned when there was one weak rating, and ‘weak’ was assigned when there were two or more weak ratings on the components of EPHPP. The quality assessment was conducted by two authors. Any discrepancy of scoring was discussed to reach consensus. Some components of EPHPP were labelled as not applicable for some studies. For example, the component of withdrawals and dropouts were not applicable for studies with retrospective study design, while that of blinding was not applicable for non-comparative studies, case series, or studies with a single group.

## 3. Results

### 3.1. Literature Search

**Figure 1:**
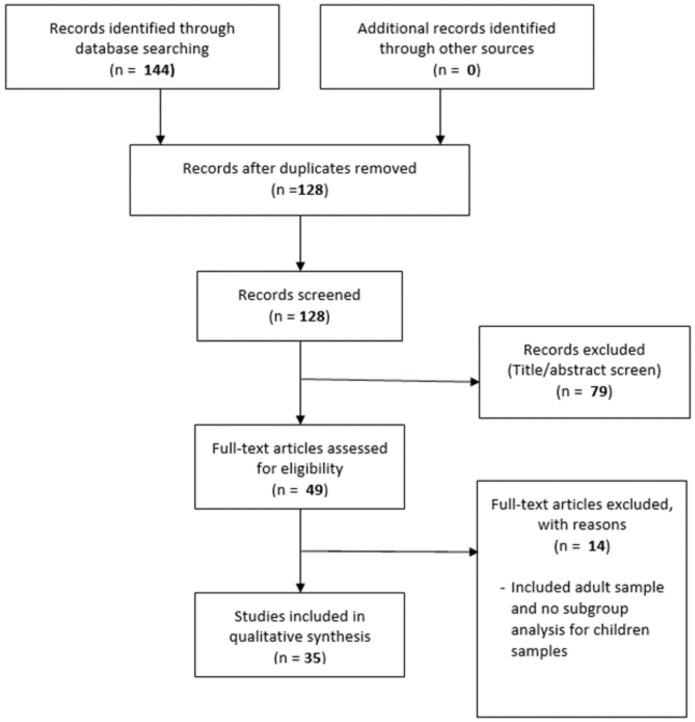
Preferred Reporting Items for Systematic Reviews and Meta-Analyses (PRISMA) flow chart.

A total of 144 articles were identified from the electronic databases. One hundred and twenty- eight articles remained after duplicates were removed. Seventy-nine articles were excluded after screening the titles and abstract as they did not meet the review criteria. Of the remaining 49 articles, data extraction was done by two authors independently; 14 articles that included adult samples and did not report subgroup analysis result for children samples below 16 years old were excluded. A total of 35 studies which fulfilled the selection criteria were included in the review (see Figure 1). From the included studies, several types of study designs were used by the authors. These were randomised controlled trials (5 studies), non-randomised controlled trials (5 studies), retrospective with comparative groups (4 studies), non-comparative or single group (20 studies), and retrospective record review with descriptive study (1 study).

### 3.2. Description of the Studies

A total of 2953 patients were pooled. The total number of patients for each study ranged from 4 to 635 patients. The mean age of patients ranged from 15 months to 11 years old. All studies involved the use of silicone stents. The majority of the included studies involved lacrimal intubation (85.7%, 30 studies), with or without a comparative group; followed by intubation as an adjunctive procedure to extDCR (2 studies), EDCR (1 study), both extDCR and EDCR (1 study), and balloon dacryoplasty (1 study). The duration of the stent placements varied, while the average follow-up after removal of stent ranged from 9 weeks to 40 months. Table 1 summarises the studies included in this systematic review.

### 3.3. Outcomes

A meta-analysis was not performed due to the heterogeneity of all the included studies. Hence, meaningful interpretation of the study outcomes in the included studies required expert discussion and clinical judgement. The two main outcomes, percentage of success and presence of complications, are narratively described in Table 2. The overall success outcome of the studies’ interventions ranged from 41.1% to 100%. Post-operative complications were reported in 23 studies, while nine studies reported no complications. Three studies did not report the complication rate.

### 3.4. Quality Assessment

Using the EPHPP global rating decision tool, four studies were assessed as being of moderate quality and 31 of weak quality (see Table 3). Most of the studies were considered weak due to the study design and non-control of confounding factors. However, based on individual methodology component assessment, all studies were assessed as being of strong quality in terms of selection bias. All eligible patients in the included studies were from hospital-based samples. As clinical cases of children with nasolacrimal duct obstruction requiring surgical intervention were limited in nature, probability sampling methods such as the random sampling method were considered infeasible. Therefore, the authors considered that the studies assessed had included samples that were representative of their target population and were thus of strong quality in terms of selection bias. Five studies were rated as strong quality in terms of study design because the authors used randomised controlled trials in their intervention study. Four studies were rated as strong quality in terms of controlling for confounding variables (e.g., age, gender), as confounders were either balanced at baseline, or controlled for during the analysis. Data collection methods were considered strong for all studies because the authors used a standard assessment criteria of success, such as complete resolution of epiphora and the dye disappearance test.

## 4. Discussion

The management of children with epiphora is challenging, not only because of the miniaturized and variable anatomy of the lacrimal drainage pathways, but also because of the lack of high quality evidence regarding the optimal treatment of NLDO in children. Probing, which involves pushing a metal wire through the punctum, canaliculi, lacrimal sac, and nasolacrimal duct into the nose, is the standard of care for congenital NLDO. Although probing is successful in uncomplicated obstructions of the distal nasolacrimal duct [50], which comprise the majority of cases in children [51], NLDO due to anatomical variations or scarred tissue, such as in Down syndrome, trauma or craniofacial malformations, is more difficult to manage [28,52,53,54,55]. In cases which fail primary probing, treatment options include repeat probing, lacrimal intubation or balloon dacryoplasty, before resorting to DCR as a last measure [6,56]. The role of stenting in the management of children with NLDO is poorly defined. Unlike in adults with acquired NLDO, where trials have shown no benefit of intubation on the 12-month success rate of endonasal DCR [57,58], the value of stenting in cases of paediatric NLDO requires further elucidation.

Most of the studies reviewed involved only lacrimal intubation, which generally had high success rates. Two RCTs compared stenting with other interventions for NLDO in children. Elsawaby et al. found no statistically significant difference in success rates between stenting and probing as a primary treatment for patients with congenital NLDO aged 6 months to 36 months [21]. Unfortunately, the lack of blinding or controlling for confounders resulted in a weak overall global rating for this study. Other non-randomized studies comparing stenting to probing as a primary procedure were likewise rated weak; Eshraghi et al. found a significantly higher success rate (73.3% vs. 48.9%) in the Masterka stent group compared with the probing group in children older than 18 months [27], while Al-Faky et al. noted a success rate of 88% for stenting and 80.3% for probing [28]. In general, intubation has been found to have an advantage over probing alone in certain groups, such as those with bilateral congenital NLDO, Down syndrome, history of acute dacryocystitis, and other causes of complex NLDO [28,59,60]. Unfortunately, beyond a certain age, the success rate of intubation as a primary or secondary procedure after failed probing appears to decline [32,55]. A RCT by Ceylan et al. observed that intubation was inferior to balloon dilation for the primary surgical treatment of congenital NLDO in children older than three years of age [20].

Two types of intubation were evaluated in the studies assessed; intubation using monocanalicular versus bicanalicular stents. The monocanalicular stent passes through a single canaliculus to the lacrimal sac and NLD, whereas the bicanalicular stent courses through both canaliculi into the sac and nasal cavity, after which the ends are tied in a loop inside the nasal cavity. Using a monocanalicular or bicanalicular stent had no statistically significant effect on outcome [18,23], although some authors prefer monocanalicular intubation for its ease of insertion and tube removal as well as a lower incidence of canalicular slit [25,26]. The duration of stenting ranged from three weeks to six months. In most studies, stents were removed after three months. It may be important to note that retention of stents for longer than 12 months has been associated with a significantly lower success rate [61]. On the contrary, early stent removal (at approximately two months) has not been shown to affect the success rate among younger children, particularly those less than two years of age [22,37,62]. In older children, higher reoperation rates are associated with stent removal prior to 4–6 weeks [37,62].

The rationale for intubation is that the stent may maintain patency of the newly-created lacrimal drainage passage by preventing the formation of granulation-related obstruction [63]. The latter may be a particular problem in children due to their anatomically narrower nasolacrimal ducts [64], elevated inflammatory tendencies, and unpredictable remodeling in response to probing-induced trauma [65], all of which may contribute to a greater risk of restenosis and failure after probing. When used as adjunctive treatment in DCR, intubation has been associated with a significantly lower incidence of operative revision [41].

Although there is a paucity of histopathological evidence of the effect of stenting in children, comparison of lacrimal sac biopsies in adults with and without silicone stents has not demonstrated any significant differences in mucosal histopathology [66]. The duration of stenting in the aforementioned study was approximately three months. A study of tear inflammatory cytokines after endoscopic endonasal DCR observed that levels of interleukin (IL)-1β, IL-2, IL-6, vascular endothelial growth factor and fibroblast growth factor-2 were higher in patients post DCR than in the control group, but rapidly returned to control group levels after stent removal [67]. This may explain the lower success rates associated with prolonged stent retention, as persistently elevated cytokines may cause sustained inflammation and fibrosis [61].

Another downside of intubation is that stent-related complications are not uncommon [68]. The most common of these is early stent dislodgement or loss, occurring in up to 50% of cases [22,23,25,26,30,38,68]. The prognostic value of this complication depends on age and the timing of tube displacement; the risk of reoperation is higher in children older than two years with stent retention of less than two months [36,62]. Stent displacement may also cause corneal abrasions [10,26,55] and ulceration [21,38]. Minor complications include those related to the lacrimal passages, such as punctal or canalicular slitting due to cheese-wiring [25,26,30,37,43] and granuloma formation [26,37,45].

This systematic review observed that lacrimal intubation is most commonly performed as a primary procedure in children with congenital NLDO, with good outcome. It is preferred over probing alone in complex NLDO [28,59,60]. Up to approximately ten years old, age is not predictive of intubation failure [32,61]. The optimal timing of stent removal is two months post insertion, although children more than two years old may require a longer duration of stenting. The main complication is stent dislodgement. Considering the technical ease of stent manipulation and high success rates, it seems reasonable to perform primary intubation in children undergoing initial probing. However, well-designed, adequately powered RCTs are required to define the role of intubation as a primary or adjunctive procedure in the surgical management of children with NLDO.

## 5. Conclusions

This systematic review identified only two RCTs evaluating the benefit of stenting over other surgical modalities in the management of children with NLDO. In the absence of high-quality evidence, the decision of whether to perform lacrimal intubation in children with NLDO requiring surgical intervention depends on clinical judgement and other low-level evidence, such as observational non-randomised trials.

## Figures and Tables

**Table 1:** Characteristics of the studies reviewed.

Study Design	First Author, Year	Country	Procedure	Number of Patients|Number of Eyes	Mean Age in mo/yr (min–max)	Gender in % (male)	Timing of Postoperative Stent Removal in d/wk/mo	Mean Follow–up in wk/mo (min–max)
Randomised controlled trials	Andalib, 2010 [18]	Iran	LI	57|70	MCI: 34.9mo	NR	3mo	NR
					(13–71mo)			
					BCI: 38.7mo			
					(14–84mo)			
	Andalib, 2014 [19]	Iran	LI	49|53	MCI: 26.25mo	NR	3mo	NR
					(13–49mo)			
					PMCI: 26.85mo (16–68mo)			
	Ceylan, 2007 [20]	Turkey	LI	20|24 (BCI)	50.8mo (36–120mo)	NR	Average 6.2 mo	NR (12mo–NR)
	Elsawaby, 2016 * [21]	Egypt	LI	27|30	14.85mo	50	At least 3wk	16wk (NR)
					(7–30mo)			
	Kominek, 2010 [22]	Czech Republic	LI	83| 95 (Group 1: 42|48; Group 2:41|47)	NR (15–30mo)	NR	Group 1: 2mo	NR (NR–6mo)
							Group 2: 5mo	
Non–randomised controlled trials	Eshraghi, 2017a [23]	Iran	LI	99|99 (MCI:52|52; BCI:47|47)	3.56yr	57.6	3mo	NR (NR–12mo)
					(1.3–10yr)			
	Fayet, 2011 ^#^ [24]	France	LI	68|68 (Group 2:6|6; Group 3:62|62)	Group 2: NR	NR	Group 2: 39d Group 3: 29d	Group 2: 14wk (3–30wk)
					(1–9yr)			
					Group 3: NR			Group 3: 16wk (3–74wk)
					(1–6yr)			
	Lee, 2012 [25]	South Korea	LI	46|60 (BCI:22|30; MCI:24|30)	BCI:23.3mo	52.2	BCI: 5–22wk	BCI: 16.4 wk (NR)
					(9–52mo)			
					MCI: 23.1mo		MCI:5–15wk	MCI: 11.6 wk (NR)
					(8–62mo)			
	Kominek, 2011 [26]	Czech Republic	LI	53|70 (BCI:24|35; MCI:29|35)	NR (10–36mo)	44.3	3–4mo	NR (NR–6mo)
	Eshraghi, 2017b [27]	Iran	LI	45|45 (LI only, study compared LI and probing)	28mo (NR)	NR	Average 9.2 wk	NR (1wk–6mo)
Retrospective with comparative groups	Al–Faky, 2012 ^$^ [28]	Iran	LI	350|454	32.6mo	46	3mo	15.3mo
					(12–132mo)			(3–108mo)
	Kaufman, 1998 ^&^ [29]	United States	LI	64|73 (Prospective:39|48	31.8mo	NR	4–6mo	NR (3–12wk)
				Retrospective:25|25)	(12–87mo)			
	Rajabi, 2016 [30]	Iran	LI	338|338 (Crawford:248|248; Monoka:52|52; Masteka:38|38)	NR	56.1	3mo	Schedule follow up 3mo
					(1–4yr)			
	Khatib, 2017 [31]	United States	LI	53|72 (complex; simple)	22mo	NR	2–3mo	14mo
					(5–65mo)			(6–29mo)
Retrospective/prospective with single group/non–comparative/consecutive cases	Okumus, 2016 [32]	Turkey	LI	30|30	10.7yr	60	Average 4.6mo	8.8mo
					(7–15yr)			(6–16mo)
	Orhan, 1997 [33]	Turkey	LI	16|18	25mo	43.8	4–7mo	12mo
					(18–48mo)			(4–24mo)
	Eshraghi, 2014 [34]	Iran	LI	44|44	3.2yr (NR)	45.5	2mo	9mo
								(6.5–13mo)
	Ali, 2013 [35]	India	ExtDCR	10|11	9.4yr	30	12–16wk	NR (3–6mo)
					(6–15yr)			
	Engel, 2007 [10]	United States	LI	635|803	18mo	45	Median of 8wk	Median of 12wk
					(6.5–103.8mo)			
	Dotan, 2015 [36]	Israel	LI	46|54	37.6mo (NR)	52.2	4–6mo	NR
	El–Essawy, 2013 [37]	Egypt	LI	192|236	21.2mo	51	3–6mo	5mo (3–16mo)
					(8–48mo)			
	Fayet, 2012 [38]	France	LI	88|110	2.4yr	NR	3wk	33.7wk (4–139wk)
					(1–8yr)			
	Casady, 2006 [7]	United States	LI	NR|7	NR	NR	3–3.5mo	NR (4–6wk)
					(12–18mo)			
	Eloy, 2009 [39]	Belgium	EDCR	8|10	4.3yr	87.5	1–3mo	10.5mo
					(8mo–9yr)			(6–15wk)
	Han, 2015 [40]	South Korea	LI	56|77	29.8mo	53.6	2–3mo	NR
					(6mo–12yr)			
	Nemet, 2008 [41]	Australia	ExtDCR/ EDCR	82|104	6.6yr (NR)	51.2	6mo	1.44yr (6mo–8yr)
	Napier, 2016 [42]	United Kingdom	LI	177|246	2.1yr (0–9.8yr)	50.4	At least 12wk	NR (6–12wk)
	Yazici, 2006 [43]	Turkey	LI	42|50	37.3mo	47.6	3mo	18.1mo
					(9mo–7yr)			(3–48mo)
	Pelit, 2009 [44]	Turkey	LI	30|34	5yr (2–10yr)	53.3	6mo	40.32mo (12–96mo)
	Yalaz, 2004 [45]	Turkey	LI	26|29	4.85yr (2–12yr)	46.2	6mo	8.3mo (6–25mo)
	Fayet, 2010a [46]	France	LI	14|18	26.2mo (14–46mo)	NR	Average of 34d	8.7wk (3–26wk)
	Pe, 1998 [47]	United States	LI	28|34	19.5mo (5mo–5yr 3mo)	39.3	2–6mo	NR (NR)
	Fayet, 2010b [48]	France	LI	4|6	33mo (30–37mo)	NR	3wk	NR (2–3mo)
	Huang 2009 [9]	Taiwan	Balloon dacryocystoplasty and LI (MCI)	25|33	3.5yr	60	4–6mo	6mo
Five year record review (descriptive study)	Abdu, 2014 [49]	Nigeria	ExtDCR	17|NA	NR (NR–15yr)	52.9	6wk	Up to 1yr

Notes. * refer to group B, the intubation group; ^#^ Group 1 was excluded (aged over 16 years); ^$^ comparison based on age groups; ^&^ comparison based on two different cohorts (prospective and retrospective groups had monocanalicular and bicanalicular silastic tube intubation respectively); MCI, monocanalicular; BCI, bicanalicular; PMCI, pushed monocanalicular; NR, not reported; d, day; wk, week; mo, month; yr, year; min, minimum; max, maximum; %, percentage; LI, lacrimal intubation; EDCR, endoscopic dacryocystorhinostomy; ExtDCR, external dacryocystorhinostomy.

**Table 2:** Summary of reported outcomes.

First Author, Year	Criteria for Successful Outcome	Overall Successful Outcome %	Post–Operative Complication
Andalib, 2010 [18]	Munk score of 0 or 1 at 3 months after tube removal	MCI: 86.2	None
		BCI: 89	
Andalib, 2014 [19]	Complete resolution of epihora at 3 months after tube removal	MCI: 90	Slit punctum in PMCI
		PMCI: 50	
Ceylan, 2007 [20]	Complete remission of epiphora at 12 months, maintained for 4 months	62.5	Ocular irritation, false lumen in the inferior meatus, iatrogenic punctal laceration
Elsawaby, 2016 [21]	Munk’s score 0 or 1 after 3 months from surgery	83.3 *	Corneal ulcer, epistaxis
Kominek, 2010 [22]	Fluorescein dye disappearance grade 0–1, corresponding to complete resolution of previous symptoms	Group 1(removal at 2 mo): 89.6	None
		Group 2 (removal at 6 mo): 91.5	
Eshraghi, 2017 [23]	Dye disappearance test grade 0–1 & complete resolution of symptom at 12 months’ follow up	MCI: 59.6	Loss of tubes
		BCI: 74.4	
Fayet, 2011 ^&^ [24]	Absence of epiphora, absence of mucous discharge	Group 2 (age 1–9 years): 100	Group 2: none
		Group 3 (age 1–6 years): 88.3	Group 3: Loss of tube, keratitis
Lee, 2012 [25]	Complete disappearance of symptoms	BCI: 93.3	Tube prolapse, punctal slitting
		MCI: 90	
Kominek, 2011 [26]	Fluorescein dye disappearance test grade 0–1 = complete resolution from symptoms	BCI: 82.86	Dislodging of tube, premature removal, loss of tube, slitting of punctum and canaliculi, granuloma pyogenicum, corneal erosion
		MCI: 88.57	
Eshraghi, 2017b [27]	Complete absence of clinical signs and symptoms of congenital nasolacrimal duct obstruction at 6 months after the procedure	73.3	Epiphora with tubes in place
Al–Faky, 2012 [28]	Normal dye disappearance test, positive Jones primary dye test	88	NR
Kaufman, 1998 [29]	Negative dye disappearance test	79	Bilateral preseptal cellulitis, migration of punctal anchor into canaliculus, corneal abrasion, corneal ulcer, premature removal of tube
Rajabi, 2016 [30]	No sign and symptom of tearing or discharge	BCI:80.2	Tube dislodging, spontaneous extrusion, corneal abrasion, punctual slitting due to cheese wiring, punctal plug migration to canaliculus
		MCI:41.1	
Khatib, 2017 [31]	Complete resolution of symptoms, negative dye disappearance test	75	Early tube loss
Okumus, 2016 [32]	Complete resolution of previous signs and symptoms and DDT grade 0–1	73.3	None
Orhan, 1997 [33]	Resolution of symptoms and previous signs	100	Epiphora with tubes in place
Eshraghi, 2014 [34]	Complete resolution or partial improvement	82.6	None
Ali, 2013 [35]	Resolution of symptoms	91	NR
Engel, 2007 [10]	Good clearance of fluorescein dye and/or absence of symptomatic testing	96	Conjunctival–corneal abrasion
Dotan, 2015 [36]	Complete resolution of all preoperative CNLDO symptoms and signs	85	Spontaneous tube loss
El–Essawy, 2013 [37]	Complete resolution of symptoms, no epiphora, no discharge, no increase tear lake	82.2	Cheesewiring of canaliculi, late postoperative granuloma formation
Fayet, 2012 [38]	Absence of symptoms after stent removal or loss	85	Keratitis, tube loss, epiphora with tubes in place
Casady, 2006 [7]	Complete resolution of symptoms	100	None
Eloy, 2009 [39]	Complete resolution of symptoms	90	Transient slight epiphora
Han, 2015 [40]	Disappearance of epiphora symptoms by a minimum of 2 months	89.6	Tube prolapse, tube loss
Nemet, 2008 [41]	Objective confirmation of free fluorescein flow to the nose	95.2	Jones tube placement
Napier, 2016 [42]	Complete resolution of symptoms and signs	91.7	NR
Yazici, 2006 [43]	Resolution of lacrimal symptoms and signs, normal tear meniscus, and in cooperative patients, normal dye disappearance test and/or patent nasolacrimal duct on irrigation at the last examination.	86	Slit punctum
Pelit, 2009 [44]	Complete resolution of previous lacrimal symptoms and signs	100	None
Yalaz, 2004 [45]	Relief from symptom and/or positive results in fluorescein dye disappearance test	93.2 (initial intubation);	Granuloma
		100 (reintubation)	
Fayet, 2010a [46]	Absence of epiphora, absence of mucous discharge	88	Mildly watery eye
Pe, 1998 [47]	Easy, uncomplicated retrieval of the Prolene guide thread during intubation; complete resolution of previous signs and symptoms and a normal result of the dye disappearance test	97	None
Fayet, 2010b [48]	Residual epiphora after ablation	100	None
Huang 2009 [9]	Complete resolution of symptoms and normal dye disappearance test	97	None
Abdu, 2014 [49]	Patent nasolacrimal duct 1 year after surgery	88	Extrusion of the tube, infection

Notes. NNR, not reported; MCI, monocanalicular; BCI, bicanalicular; PMCI, pushed monocanalicular; * study consisted of probing and stent groups, the value refers to stent group; ^&^ study consisted of three groups; group 1 aged 44–77 years was excluded.

**Table 3:** EPHPP quality assessment tool rating for individual studies.

First Author, Year	Selection Bias	Study Design	Confounders	Blinding	Data Collection Methods	Withdrawals and Dropouts	Global Rating
Andalib, 2010 [18]	S	S	S	W	S	S	M
Andalib, 2014 [19]	S	S	S	W	S	M	M
Ceylan, 2007 [20]	S	S	W	W	S	S	W
Elsawaby, 2016 [21]	S	S	W	W	S	S	W
Kominek, 2010 [22]	S	S	W	W	S	S	W
Eshraghi, 2017 [23]	S	M	W	W	S	S	W
Fayet, 2011 [24]	S	M	W	W	S	S	W
Lee, 2012 [25]	S	M	S	W	S	S	M
Kominek, 2011 [26]	S	M	W	W	S	S	W
Eshraghi, 2017b [27]	S	M	W	W	S	S	W
Al-Faky, 2012 [28]	S	M	W	W	S	NA	W
Kaufman, 1998 [29]	S	M	W	W	S	S	W
Rajabi, 2016 [30]	S	M	W	W	S	NA	W
Khatib, 2017 [31]	S	M	W	W	S	NA	W
Okumus, 2016 [32]	S	W	W	NA	S	S	W
Orhan, 1997 [33]	S	W	W	NA	S	S	W
Eshraghi, 2014 [34]	S	W	S	NA	S	S	M
Ali, 2013 [35]	S	W	W	NA	S	S	W
Engel, 2007 [10]	S	W	W	NA	S	NA	W
Dotan, 2015 [36]	S	W	W	NA	S	NA	W
El-Essawy, 2013 [37]	S	W	W	NA	S	NA	W
Fayet, 2012 [38]	S	W	W	NA	S	NA	W
Casady, 2006 [7]	S	W	W	NA	S	NA	W
Eloy, 2009 [39]	S	W	W	NA	S	NA	W
Han, 2015 [40]	S	W	W	NA	S	NA	W
Nemet, 2008 [41]	S	W	W	NA	S	NA	W
Napier, 2016 [42]	S	W	W	NA	S	NA	W
Yazici, 2006 [43]	S	W	W	NA	S	NA	W
Pelit, 2009 [44]	S	W	W	NA	S	NA	W
Yalaz, 2004 [45]	S	W	W	NA	S	NA	W
Fayet, 2010a [46]	S	W	W	NA	S	NA	W
Pe, 1998 [47]	S	W	W	NA	S	NA	W
Fayet, 2010b [48]	S	W	W	NA	S	NA	W
Huang 2009 [9]	S	W	W	NA	S	NA	W
Abdu, 2014 [49]	S	W	W	NA	S	NA	W

Notes. EPHPP: Effective Public Health Practice Project; S: strong; M: medium; W: weak; NA: not applicable.
